# Development of a multi-phase CT-based radiomics model to differentiate heterotopic pancreas from gastrointestinal stromal tumor

**DOI:** 10.1186/s12880-024-01219-2

**Published:** 2024-02-14

**Authors:** Kui Sun, Shuxia Yu, Ying Wang, Rongze Jia, Rongchao Shi, Changhu Liang, Ximing Wang, Haiyan Wang

**Affiliations:** 1 Department of General Surgery, Peking University Third Hospital, 49 North Garden Road, Haidian District, 100191 Beijing, China; 2 Department of Radiology, Shandong Provincial Hospital Affiliated to Shandong First Medical University, Jing Wu Road, NO. 324, 250021 Jinan, Shandong China; 3 Department of Gastroenterology, Shandong Provincial Hospital Affiliated to Shandong First Medical University, Jing Wu Road, No. 324, 250021 Jinan, China; 4 Department of Radiology, Affiliated Hospital of Shandong University of Traditional Chinese Medicine, Shandong Provincial Hospital of Traditional Chinese Medicine, Jing Shi Road, No. 16369, 250014 Jinan, China; 5 Department of Radiology, Shandong Provincial Hospital, Shandong University, Jing Wu Road, No. 324, 250021 Jinan, China

**Keywords:** Heterotopic pancreas, Gastrointestinal stromal tumor, CT, Radiomics

## Abstract

**Background:**

To investigate whether CT-based radiomics can effectively differentiate between heterotopic pancreas (HP) and gastrointestinal stromal tumor (GIST), and whether different resampling methods can affect the model’s performance.

**Methods:**

Multi-phase CT radiological data were retrospectively collected from 94 patients. Of these, 40 with HP and 54 with GISTs were enrolled between April 2017 and November 2021. One experienced radiologist manually delineated the volume of interest and then resampled the voxel size of the images to 0.5 × 0.5 × 0.5 mm^3^, 1 × 1 × 1 mm^3^, and 2 × 2 × 2 mm^3^, respectively. Radiomics features were extracted using PyRadiomics, resulting in 1218 features from each phase image. The datasets were randomly divided into training set (*n* = 66) and validation set (*n* = 28) at a 7:3 ratio. After applying multiple feature selection methods, the optimal features were screened. Radial basis kernel function-based support vector machine (RBF-SVM) was used as the classifier, and model performance was evaluated using the area under the receiver operating curve (AUC) analysis, as well as accuracy, sensitivity, and specificity.

**Results:**

The combined phase model performed better than the other phase models, and the resampling method of 0.5 × 0.5 × 0.5 mm^3^ achieved the highest performance with an AUC of 0.953 (0.881-1), accuracy of 0.929, sensitivity of 0.938, and specificity of 0.917 in the validation set. The Delong test showed no significant difference in AUCs among the three resampling methods, with *p* > 0.05.

**Conclusions:**

Radiomics can effectively differentiate between HP and GISTs on CT images, and the diagnostic performance of radiomics is minimally affected by different resampling methods.

**Supplementary Information:**

The online version contains supplementary material available at 10.1186/s12880-024-01219-2.

## Introduction

Heterotopic pancreas (HP) is a congenital anomaly in which pancreatic tissue is separate from the main gland and lacks a continuous vascular or ductal connection [1]. HP is most commonly found in the upper gastrointestinal tract, such as the stomach, duodenum, and proximal jejunum, but is rare in other locations such as the esophagus, ileum, and biliary tree. The majority of affected patients are asymptomatic, making HP difficult to diagnose in clinical settings. The lesion is typically discovered incidentally during an unrelated surgery, imaging examination, or at autopsy. Autopsy studies [2–3] have reported an incidence of 0.5-13.7%, while the incidence during upper abdominal surgeries and gastrectomies is 0.2% and 0.9%, respectively.

HP often presents as a submucosal mass of the gastrointestinal tract and can be misdiagnosed as other submucosal tumors, particularly gastrointestinal stromal tumors (GISTs), in medical imaging or endoscopy. GISTs are specific mesenchymal tumors that can develop in different locations throughout the gastrointestinal tract, omentum, and mesentery [4–5]. GISTs are invasive and potentially malignant, with a 20-30% risk of malignancy and poor prognosis when they give rise to abdominal cavities or liver metastases [6–7]. Resection is the only curative treatment for GISTs. In contrast, HP generally does not require treatment unless complications occur.

Computed tomography (CT) is the ideal imaging modality for upper gastrointestinal examination and preoperative evaluation, but the similar appearance of HP and GISTs in CT scans poses a diagnostic challenge. The characteristic radiographic appearance of HP is a small broad-based submucosal mass in the antrum with ill-defined or microlobulated margins, resembling leiomyoma or other submucosal tumors such as GISTs [8–10]. HP generally shows obvious enhancement in the late arterial phase and a gradual decrease in the venous and delayed phases on enhanced CT. Lesions in small GISTs exhibits uniform enhancement on enhanced CT. The venous phase enhancement is slightly higher than that in the arterial phase, but detecting changes in GISTs is challenging with the naked eye. As a result, partial patients with HP have received unnecessary operative resection by being misdiagnosed as having GISTs.

Radiomics, first proposed by Lambin et al. [11], allows for in-depth tumor phenotyping and quantification of lesion heterogeneity, providing high-throughput quantitative data from radiological images that are invisible to the human eye. Several studies have explored the potential value of radiomics in abdominal oncology, with promising results for lesion characterization, assessment of therapeutic response, and patient survival [12–14]. The multi-phase enhanced CT images show similarities between HP and GISTs, making it difficult for radiologists to visually differentiate. Nevertheless, potential subtle differences in shape, intensity, and texture might be emerging, hinting at the suitability of radiomics approach.

Therefore, this study aims to develop and validate a CT-based radiomics model that can discriminate between HP and GISTs, and to investigate the effect of different resampling parameters on the model’s performance.

## Materials and methods

### Patients

**Fig. 1:**
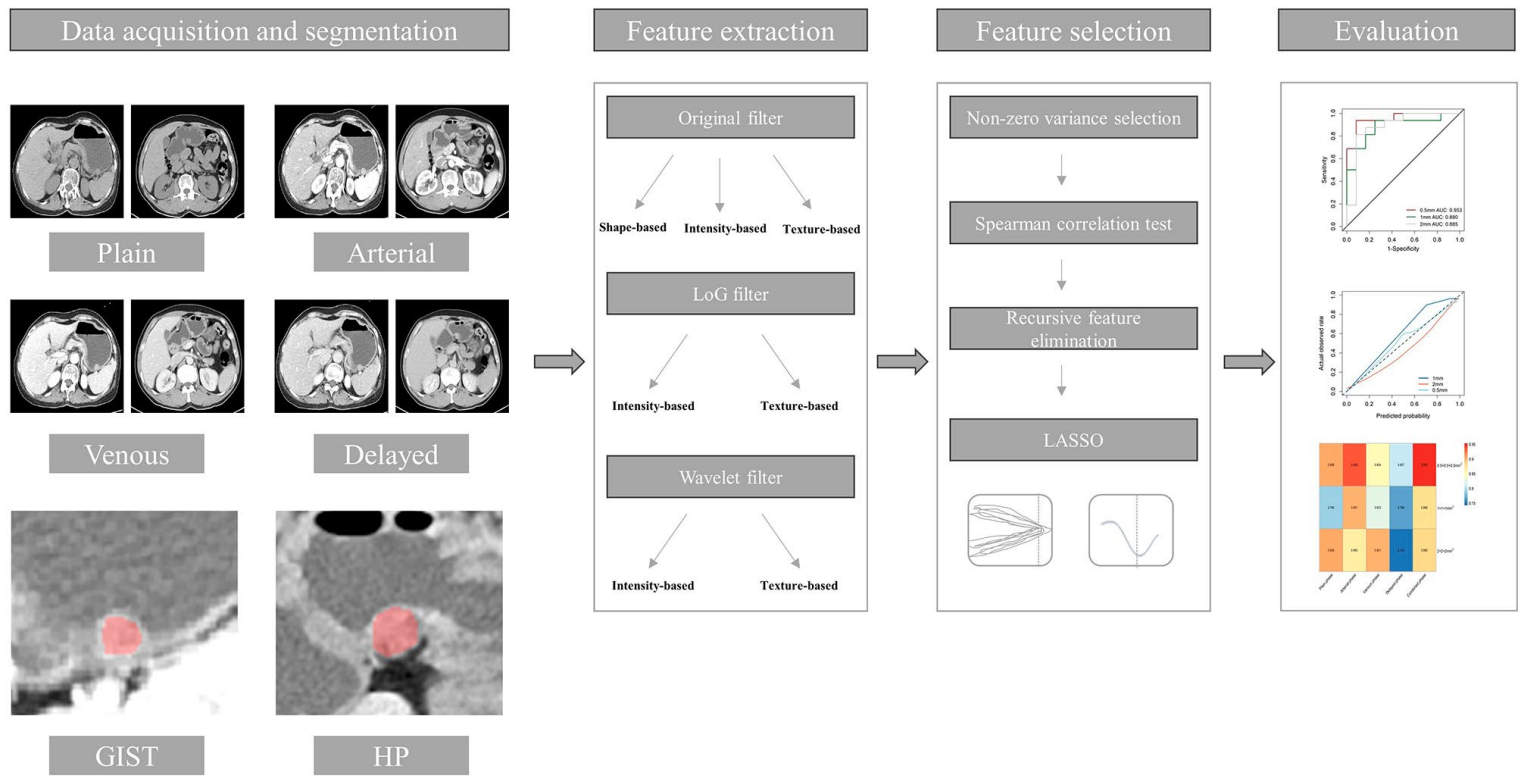
Flowchart of the analysis in this study

A total of 94 patients who underwent abdominal CT examinations were included in this study. Among them, 40 with HP and 54 with GISTs were enrolled from April 2017 to October 2021 at single center. The inclusion criteria is as follow: (1) all patients were proved HP or GISTs by pathology after resection. (2) diameters of GISTs lesions were less than 3 cm. (3) all patients underwent abdominal multi-phase CT scan, including: CT plain, arterial, venous and delayed phases. The exclusion criteria is as follow: (1) patients with incomplete clinic-pathological data. (2) poor quality CT scan, including: blurred lesion boundaries and severe artifacts. (3) lack one of the multi-phase CT scan. The retrospective study was approved by our institutional review board, and the need to obtain informed consent was waived. The whole analysis workflow in this study is shown in Fig. 1.

### CT protocol

The CT examinations were performed using Siemens Force dual-source 128-row CT scanner and Siemens Definition Flash dual-source 128-row CT scanner with the following parameters: 100mAs tube current, 120 kV tube voltage, 512 × 512 matrix, 1.5 pitch, 1.5 mm layer thickness, and 1 mm layer spacing. Intravenous group injection tracking method was used for contrast agent enhancement, with iodophoresis injection (Shanghai Stellite) selected as the contrast agent, which had an iodine concentration of 350 mg/mL, dose of 80mL, and flow rate of 23mL/s. The scan was delayed for 30, 60, 90, 120s for the arterial phase, venous phase, and delayed phase after the injection was completed.

### CT radiomics analysis

One radiologist, Rz.J, with 13 years of experience in abdominal CT manually delineated the volume of interest (VOI) using TIK-SNAP software (version 3.8.0). The VOI covered the entire lesion and excluded the equivocal boundary of the mass. All segmentations were confirmed by another radiologist, Hy.W, with 25 years of experience in abdominal CT. Appropriate adjustments were made after a consensus-based discussion when disagreements arose.

Radiomics was implemented using PyRadiomics, an open-source Python tool package (3.0.1). Prior to this, the voxel size of the images was resampled to 0.5 × 0.5 × 0.5 mm^3^, 1 × 1 × 1 mm^3^, and 2 × 2 × 2 mm^3^, respectively. Gray-level was discretized using a fixed bin width value of 70 with absolute discretization from − 1000 to 3000 Hounsfield units. Radiomics feature extraction was performed separately for the four-phase images (plain, arterial, venous, and delayed phases). Features were extracted from the original image and filtered images, which included Laplacian of Gaussian (LoG) and wavelet filtering. Eventually, 1218 features, including geometry-based, grayscale-based, and texture-based were acquired from each phase image.

### Radiomics feature selection and classifier building

The patients were randomly divided into a training set (n = 66) and a validation set (n = 28) at a 7:3 ratio. Prior to feature selection, z-score normalization was applied to the training set. Firstly, predictors with zero variance were removed. Secondly, high-correlational variables with rs > 0.8 were eliminated using the Spearman test. Thirdly, recursive feature elimination (RFE) with a treebagging algorithm was used to analyze the variables, and those with an ‘accuracy’ index were retained via five-fold cross-validation. Lastly, the optimal predictors with non-multicollinearity and anti-overfitting were selected using the least absolute shrinkage and selection operator (LASSO) regression model. These features were preserved via the one standard error of the minimum lambda (λ) of five-fold cross-validation.

Support vector machine (SVM) has several advantages such as good performance on small-scale datasets, rapid computational speed, multiple optional kernels, well generalization, and achieving an optimal global solution with convex optimization problems [15]. In this study, the radial basis kernel function-based SVM (RBF-SVM) was chosen as the main model for analysis. Each phase RBF-SVM model of different resampling methods was constructed to evaluate the diagnostic performance. Combined phase model was constructed by optimal features of each phase via final LASSO selection method.

### Statistical analysis

The difference in categorical variables was calculated using the Chi-Square method, while the difference in continuous variables was calculated using t-test or Mann-Whitney U test and described as mean ± standard deviation or median (IQR). The diagnostic performance of radiomics features was evaluated using the area under the receiver operating curve (AUC) analysis, as well as accuracy, sensitivity, and specificity. The AUC values among different phase models were compared using the DeLong method, and a *p*-value of less than 0.05 was considered statistically significant.

## Results

### Baseline characteristic

Out of the 94 patients included in the study, 40 had HP and 54 had GIST. The average age of the HP patients was 47.8 ± 14.0 years (ranging from 20 to 71 years), with 23 males and 17 females. The average age of the GIST patients was 58.7 ± 8.3 years (ranging from 41 to 77 years), with 22 males and 32 females. The HP patients in this study tended to be younger than those with GIST. Further details regarding the baseline information can be found in Table 1.

**Table 1:** The baseline characteristics of patients

Baseline characteristics	Heterotopic pancreas (HP)	gastrointestinal stromal tumor (GIST)
Number of participants	40	54
Age (years)	47 ± 14	59 ± 8
Gender		
Man	23 (57.5%)	22 (40.7)
Woman	17 (42.5%)	32 (59.3)
Resection type		
Gastrectomy under laparoscopic surgery	9 (22.5%)	6 (11.1%)
Conventional gastrectomy	3 (7.5%)	4 (7.4%)
Gastrointestinal endoscopic surgery	28 (70%)	44 (81.5%)
Size (cm)		
Length diameter	0.9 − 0.35	0.7 − 0.3
Short diameter	0.5–2.7	0.6–2.4
Location		
Proximal stomach	2 (5%)	30 (55.6%)
Middle stomach	21 (52.5%)	19 (35.2%)
Distal stomach	17 (42.5%)	5 (9.2%)

### Discrimination performance and feature contribution

**Fig. 2:**
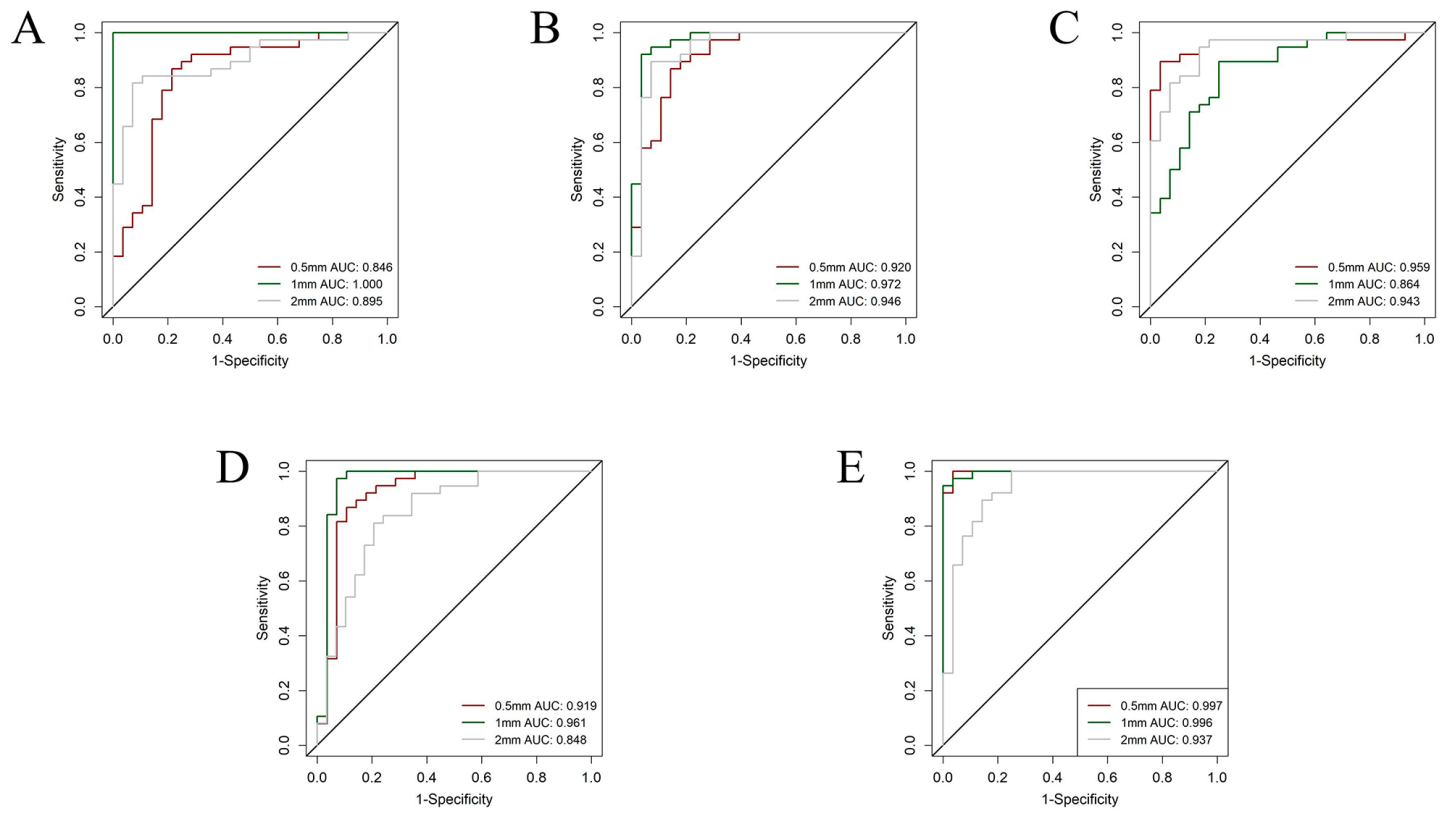
Different resampling method auc performance in plain phase (**A**), arterial phase (**B**), venous phase (**C**), delayed phase (**D**) and combined phase (**E**) on the training set

All classifiers using different resampling methods showed favorable performance, with the combined phase model performing better than the other phase models. In the resampling of 0.5 × 0.5 × 0.5 mm^3^ radiomics, the combined phase model achieved an AUC of 0.997 (0.991-1), accuracy of 0.985, sensitivity of 1, and specificity of 0.964 in the training set (Fig. 2). It also achieved an AUC of 0.953 (0.881-1), accuracy of 0.929, sensitivity of 0.938, and specificity of 0.917 in the validation set. The combined phase RBF-SVM comprised of 4 original and 4 wavelet filter features. Out of these, 2 were shape statistics, 2 were first-order statistics, and 4 were textural statistics.

In resampling of 1 × 1 × 1 mm^3^ radiomics, the combined phase model was the best performer with an AUC of 0.996 (0.989-1), accuracy of 0.970, sensitivity of 0.947, and specificity of 1 in the training set. In the validation set, it achieved an AUC of 0.880 (0.748-1), accuracy of 0.857, sensitivity of 0.938, and specificity of 0.750. This model included 4 original, 1 LoG, and 5 wavelet filter features. Out of these, 2 were shape statistics, 1 was first-order statistics, and 7 were textural statistics.

**Fig. 3:**
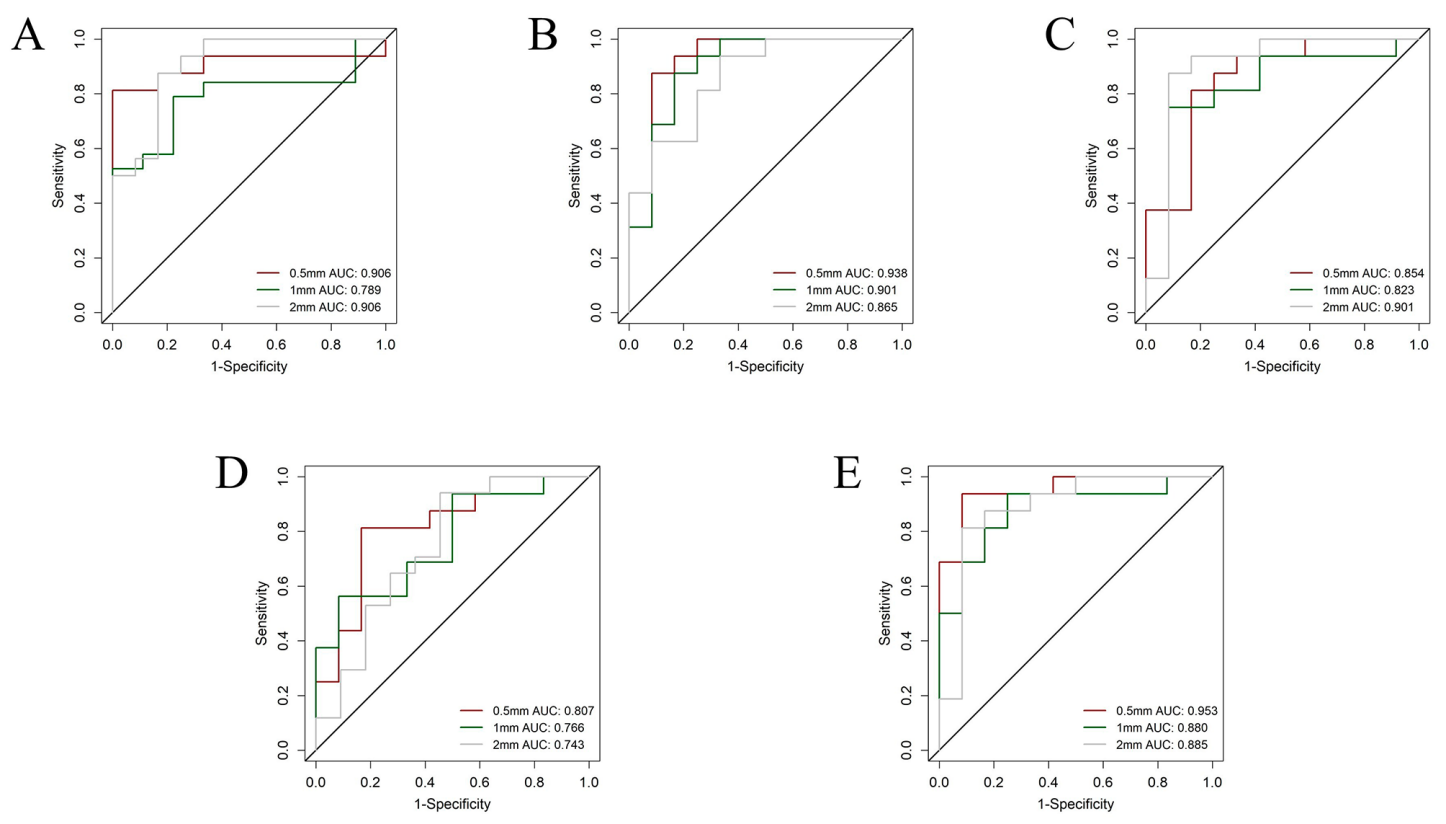
Different resampling method auc performance in plain phase (**A**), arterial phase (**B**), venous phase (**C**), delayed phase (**D**) and combined phase (**E**) on the validation set

In the resampling of 2 × 2 × 2 mm^3^ radiomics, the venous phase model was the best performer with an AUC of 0.943 (0.889–0.996), accuracy of 0.894, sensitivity of 0.947, and specificity of 0.821 in the training set. It achieved an AUC of 0.901 (0.754-1), accuracy of 0.893, sensitivity of 0.875, and specificity of 0.917 in the validation set (Fig. 3). The venous phase RBF-SVM contained 2 original, 1 LoG, and 4 wavelet filter features. Out of these, 1 was shape statistics, 2 were first-order statistics, and 4 were textural statistics. The performance of each phase model is shown in Table 2.

**Table 2:** The diagnostic performance of each model in training and validation sets

	Training set					Validation set			
	AUC	ACC	SEN	SPE		AUC	ACC	SEN	SPE
Plain phase									
0.5 mm	0.846 (0.743–0.949)	0.833	0.868	0.786		0.906 (0.777-1)	0.893	0.813	1.000
1 mm	1 (1–1)	1.000	1.000	1.000		0.789 (0.617–0.963)	0.786	0.789	0.778
2 mm	0.895 (0.818–0.972)	0.864	0.816	0.929		0.906 (0.791-1)	0.857	0.875	0.833
Arterial phase									
0.5 mm	0.920 (0.851–0.989)	0.864	0.868	0.857		0.939 (0.839-1)	0.893	0.875	0.917
1 mm	0.972 (0.931-1)	0.939	0.921	0.964		0.901 (0.775-1)	0.857	0.875	0.833
2 mm	0.946 (0.884-1)	0.909	0.895	0.929		0.865 (0.729-1)	0.821	0.938	0.667
Venous phase									
0.5 mm	0.959 (0.907-1)	0.924	0.895	0.964		0.854 (0.702-1)	0.821	0.813	0.833
1 mm	0.864 (0.775–0.952)	0.833	0.895	0.750		0.823 (0.648–0.998)	0.821	0.750	0.917
2 mm	0.943 (0.889–0.996)	0.894	0.947	0.821		0.901 (0.754-1)	0.893	0.875	0.917
Delayed phase									
0.5 mm	0.919 (0.838-1)	0.879	0.868	0.893		0.807 (0.635–0.980)	0.821	0.813	0.833
1 mm	0.962 (0.898-1)	0.955	0.974	0.929		0.766 (0.587–0.944)	0.714	0.563	0.917
2 mm	0.848 (0.750–0.946)	0.803	0.811	0.793		0.743 (0.537–0.949)	0.786	0.941	0.545
Combined phase									
0.5 mm	0.997 (0.991-1)	0.985	1.000	0.964		0.953 (0.881-1)	0.929	0.938	0.917
1 mm	0.996 (0.989-1)	0.970	0.947	1.000		0.880 (0.748-1)	0.857	0.938	0.750
2 mm	0.937 (0.875–0.999)	0.879	0.895	0.857		0.885 (0.741-1)	0.857	0.812	0.917

AUC, the area under the receiver operating characteristic curve; ACC, accuracy; SEN, sensitivity; SPE, specificity

Based on these results, the globally optimal model was the combined phase model originating from the resampling of 0.5 × 0.5 × 0.5 mm^3^ radiomics, and the optimal hyperparameters are “C” of 0.4 and “gamma” of 1.

### AUCs comparison among different resampling methods

**Fig. 4:**
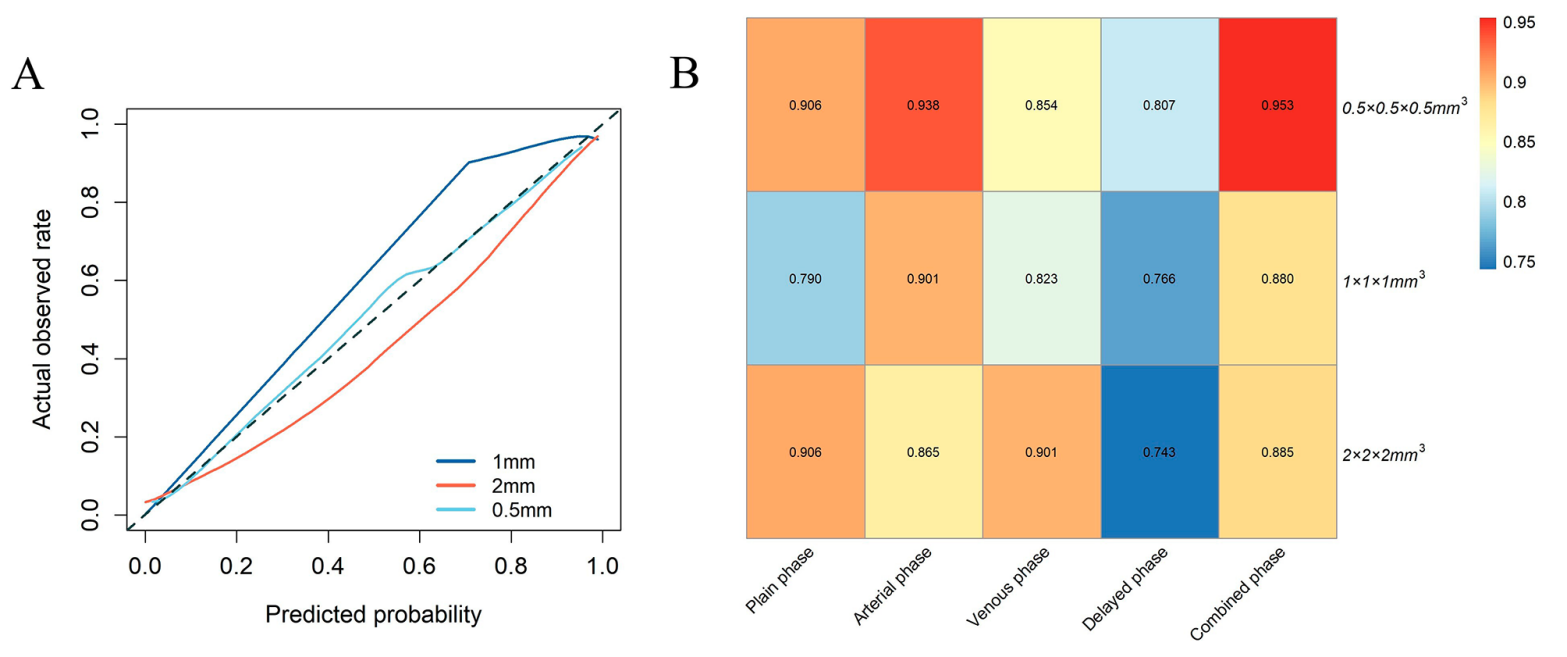
Between the predicted and true values. Calibration curves for the best model from 0.5 mm, 1 and 2 mm (**A**) resampling methods. Heatmap of the auc performance among different resampling methods and phases (**B**)

Although the models showed similar diagnostic efficacy on different resampling methods, we used the DeLong method to assess whether there were any differences among the AUCs of the models on the validation set. The results showed no significant difference in AUCs, with *p*-values of 0.177, 0.275 and 0.951 in the combined phase, and 0.295, 1, and 0.276 in the plain phase, respectively. The comparison of the other phases is shown in Table 3. Figure 4 depicts the calibration curve for the best model in different resampling methods and heatmap of the AUCs performance of the different resampling methods and phases. The names of the selected features in each phase model are provided in the supplementary materials.

**Table 3:** The auc comparison of different resampling method using Delong method in validation set

Scan phases	Resampling	AUC	P
Plain			
	0.5 mm vs. 1 mm	0.91 vs. 0.79	0.295
	0.5 mm vs. 2 mm	0.91 vs. 0.91	1
	1 mm vs. 2 mm	0.79 vs. 0.91	0.276
Arterial			
	0.5 mm vs. 1 mm	0.94 vs. 0.90	0.379
	0.5 mm vs. 2 mm	0.94 vs. 0.86	0.321
	1 mm vs. 2 mm	0.90 vs. 0.86	0.632
Venous			
	0.5 mm vs. 1 mm	0.85 vs. 0.82	0.659
	0.5 mm vs. 2 mm	0.85 vs. 0.90	0.517
	1 mm vs. 2 mm	0.82 vs. 0.90	0.465
Delayed			
	0.5 mm vs. 1 mm	0.81 vs. 0.77	0.694
	0.5 mm vs. 2 mm	0.81 vs. 0.74	0.643
	1 mm vs. 2 mm	0.77 vs. 0.74	0.873
Combined			
	0.5 mm vs. 1 mm	0.95 vs. 0.88	0.177
	0.5 mm vs. 2 mm	0.95 vs. 0.89	0.275
	1 mm vs. 2 mm	0.88 vs. 0.89	0.951

### Overlap radiomics features

There were overlapping radiomics features across different resampling methods on the plain, arterial, and venous phases. In the plain phase, the overlapping feature was the first-order statistic ‘Median’. In the arterial phase, the overlapping feature was the shape statistic ‘Sphericity’. In the venous phase, the overlapping features were the first-order statistic ‘Mean’ and the shape statistic ‘Sphericity’. All these features were derived from the original filter.

## Discussion

There are few studies that focus on radiomics to differentiate between HP and GISTs on CT scans. Our study shows that a CT-based radiomics model can be used to distinguish between HP and GISTs, and that different resampling methods do not affect the diagnostic performance of the models. There was no significant difference in the performance of each phase model (*p* > 0.05). In the resampling of 0.5 × 0.5 × 0.5 mm^3^, the best model is from combined phase, achieved the best performance with AUC of 0.953. In the resampling of 1 × 1 × 1 mm^3^, the best model (combined phase) achieved the best performance with AUC of 0.880. In the resampling of 2 × 2 × 2 mm^3^, the best model (venous phase) achieved the best performance with AUC of 0.901.

The contrast-enhanced CT is the most applicable imaging modality in such diseases, and the imaging presentation of HP correlates with its histological components. HP is often presented as a well-defined intramural ovoid mass on CT images, with a diameter of less than 3 cm. Lesions consisting mainly of acinar tissues tend to have a higher or equal degree of enhancement than the pancreas in situ, whereas lesions consisting mainly of ductal structures and hyperplastic muscular layers tend to have a lower degree of enhancement than the normal pancreas. However, both lesions of HP and GISTs are presented with homogeneous enhancement. In our study, except for the combination phase, the arterial phase model achieved the best performance compared to other phases. The mean AUC in different resampling methods was 0.901 on the validation set, and the remaining phases were 0.867, 0.859, and 0.772, respectively.

As a branch of artificial intelligence (AI), radiomics has become one of the most popular research fields in medical imaging. When radiomics is combined with machine learning, it can exert the best efficacy of a model. Although radiomics has a long way to go before it can be applied in reality, there are some studies that demonstrate its potential value [16–19]. The essence of radiomics is a large pile of radiomics features, and the feature values between different classes can be mined and analyzed to reflect the status of a disease.

There are several overlapping features across the different resampling methods, with the feature ‘Sphericity’ occupying 50% (2/4). ‘Sphericity’ is defined as a measure of the roundness of the shape of the tumor region relative to a sphere, where a value of 1 indicates a perfect sphere. In our analysis, the values of GISTs were found to be significantly higher than those of HP in the arterial phase (0.5 × 0.5 × 0.5 mm^3^: 0.730 (0.714–0.759) vs 0.696 (0.663–0.718) [p < 0.001]; 1 × 1 × 1 mm^3^: 0.778 (0.757–0.810) vs 0.741 (0.706–0.774) [p < 0.001]; 2 × 2 × 2 mm^3^: 0.815 (0.772–0.841) vs 0.776 (0.742–0.796) [p = 0.001]), as well as in the venous phase (0.5 × 0.5 × 0.5 mm^3^: 0.724 (0.703–0.750) vs 0.701 (0.660–0.731) [p = 0.005]; 1 × 1 × 1 mm^3^: 0.765 (0.745-0.800) vs 0.744 (0.703–0.770) [p = 0.008]; 2 × 2 × 2 mm^3^: 0.807 (0.768–0.847) vs 0.792 (0.737–0.820) [p = 0.024]). This study is consistent with Jang’s report [20]. GISTs are composed of spindle cells, epithelioid cells, or a mixture, and are more likely to be round in shape than HP, which commonly exhibits a slender appearance similar to that of a normal pancreas. According to the report by Li et al. [21], they found that GISTs (42%) were significantly rounder than HP masses (8%). However, this phenomenon disagrees with Yang’s research [22]. For the overlapping feature ‘Median’ in the plain phase, the value in the HP group is higher than that in the GISTs group in resampling of 0.5 × 0.5 × 0.5 mm^3^ (42.5 (39.5–48) vs 33 (28–38) [p < 0.001]), 1 × 1 × 1 mm^3^ (43 (39.25–48.25) vs 33 (28–39) [p < 0.001]), and 2 × 2 × 2 mm^3^ (43 (39–48) vs 33 (27.250-39.625) [p < 0.001]). For the overlapping feature ‘Mean’ in the venous phase, the value in the HP group is also higher than that in the GISTs group in resampling of 0.5 × 0.5 × 0.5 mm^3^ (86.141 (74.459–102.600) vs 68.975 (61.223–78.842) [*p* < 0.001]), 1 × 1 × 1 mm^3^ (85.901 (75.048-103.251) vs 70.538 (61.293–79.036) [*p* < 0.001]), and 2 × 2 × 2 mm^3^ (83.729 (73.346-103.957) vs 70.349 (59.756–77.962) [*p* < 0.001]).

Our study has several limitations. First, the use of multiple CT scanners in this retrospective study resulted in nonconformity of the scanning parameters and volumes of contrast media. To address this issue, we normalized the grey level and resampled the voxel spacing to eliminate the possible adverse effects. Second, the VOI was manually delineated by radiologists, which is time-consuming and subject to some degree of subjectivity. Third, the sample size is small, a prospective, multi-center, and large-scale population is still necessary for further validation., a prospective, multi-center, and large-scale population is still necessary for further validation.

## Conclusions

Radiomics can serve as a noninvasive and quantitative imaging biomarker to differentiate between HP and GISTs on CT images, thereby providing clinical guidance to radiologists in decision-making. Furthermore, this study suggests that the diagnostic performance of radiomics is minimally affected by different resampling methods.

### Electronic supplementary material

Below is the link to the electronic supplementary material.

Supplementary Material 1

## Data Availability

The datasets used and/or analysed during the current study are available from the corresponding author on reasonable request.

## Abbreviations

HP: Heterotopic pancreas
GIST: Gastrointestinal stromal tumor
VOI: Volume of interest
LoG: Laplacian of Gaussian
RFE: Recursive feature elimination
LASSO: The least absolute shrinkage and selection operator
RBF-SVM: Radial basis kernel function-based support vector machine
AUC: The area under the receiver operating characteristic curve
AI: Artificial intelligence
