# Supplementary material for: Development of a multi-phase CT-based radiomics model to differentiate heterotopic pancreas from gastrointestinal stromal tumor
Source: BMC Med Imaging. 2024 Feb 14;24:44. doi: 10.1186/s12880-024-01219-2 (PMC10868069; doi:10.1186/s12880-024-01219-2)
Supplement: Supplementary file 1 — Supplementary Material 1 [file 12880_2024_1219_MOESM1_ESM.docx]

**Stable 1. The names of the selected features in each phase model.**

| **Resampling methods** | **Phase (number)** | **Feature name** |
| --- | --- | --- |
| 0.5×0.5×0.5mm^3^ | Plain (2) | original_firstorder_Median |
|  |  | wavelet.LLH_glrlm_RunEntropy |
|  | Arterial (6) | original_firstorder_Median |
|  |  | original_shape_Sphericity |
|  |  | wavelet.LHH_firstorder_Skewness |
|  |  | wavelet.LLL_glszm_SmallAreaEmphasis |
|  |  | log.sigma.3.0.mm.3D_glszm_SmallAreaEmphasis |
|  |  | wavelet.LLH_glrlm_ShortRunEmphasis |
|  | Venous (6) | original_shape_Sphericity |
|  |  | original_firstorder_Mean |
|  |  | wavelet.LLH_glrlm_ShortRunLowGrayLevelEmphasis |
|  |  | original_glcm_MaximumProbability |
|  |  | log.sigma.5.0.mm.3D_gldm_LargeDependenceLowGrayLevelEmphasis |
|  |  | wavelet.LHH_firstorder_Kurtosis |
|  | Delayed (6) | wavelet.LLH_glrlm_ShortRunEmphasis |
|  |  | wavelet.LLH_glrlm_LongRunEmphasis |
|  |  | original_shape_Sphericity |
|  |  | wavelet.LLL_glrlm_LongRunLowGrayLevelEmphasis |
|  |  | original_shape_Flatness |
|  |  | wavelet.LLL_glszm_GrayLevelNonUniformityNormalized |
|  | Combined (8) | wavelet-LLH_glrlm_ShortRunLowGrayLevelEmphasis_v |
|  |  | original_shape_Sphericity_a |
|  |  | original_firstorder_Mean_v |
|  |  | wavelet-LLH_glrlm_ShortRunEmphasis_d |
|  |  | wavelet-LLL_glszm_GrayLevelNonUniformityNormalized_d |
|  |  | original_glcm_MaximumProbability_v |
|  |  | original_shape_Sphericity_d |
|  |  | wavelet-LHH_firstorder_Skewness_a |
| 1×1×1mm^3^ | Plain (6) | original_firstorder_Median |
|  |  | wavelet.LHL_glszm_SizeZoneNonUniformityNormalized |
|  |  | wavelet.LLH_glrlm_ShortRunLowGrayLevelEmphasis |
|  |  | wavelet.LLH_glcm_Correlation |
|  |  | log.sigma.2.0.mm.3D_glcm_Imc1 |
|  |  | wavelet.HLL_firstorder_RootMeanSquared |
|  | Arterial (4) | original_firstorder_Median |
|  |  | original_shape_Sphericity |
|  |  | log.sigma.2.0.mm.3D_glszm_SmallAreaLowGrayLevelEmphasis |
|  |  | wavelet.LLH_glszm_ZoneEntropy |
|  | Venous (4) | original_shape_Sphericity |
|  |  | original_firstorder_Mean |
|  |  | log.sigma.1.0.mm.3D_glszm_GrayLevelNonUniformity |
|  |  | original_glcm_MaximumProbability |
|  | Delayed (7) | original_glcm_Imc1 |
|  |  | original_shape_Sphericity |
|  |  | wavelet.LLH_glcm_Correlation |
|  |  | wavelet.LLL_gldm_LargeDependenceLowGrayLevelEmphasis |
|  |  | wavelet.HLL_glszm_SizeZoneNonUniformityNormalized |
|  |  | wavelet.LLL_glszm_SizeZoneNonUniformityNormalized |
|  |  | wavelet.HLH_glszm_GrayLevelNonUniformityNormalized |
|  | Combined (10) | original_firstorder_Mean_v |
|  |  | original_shape_Sphericity_a |
|  |  | wavelet-LLL_gldm_LargeDependenceLowGrayLevelEmphasis_d |
|  |  | log-sigma-1-0-mm-3D_glszm_GrayLevelNonUniformity_v |
|  |  | wavelet-LLH_glcm_Correlation_d |
|  |  | wavelet-LLH_glszm_ZoneEntropy_a |
|  |  | original_glcm_Imc1_d |
|  |  | wavelet-LLH_glrlm_ShortRunLowGrayLevelEmphasis |
|  |  | original_shape_Sphericity_d |
|  |  | wavelet-LHL_glszm_SizeZoneNonUniformityNormalized |
| 2×2×2mm^3^ | Plain (5) | original_firstorder_Median |
|  |  | log.sigma.4.0.mm.3D_glcm_Correlation |
|  |  | original_shape_Sphericity |
|  |  | wavelet.LLH_firstorder_10Percentile |
|  |  | log.sigma.1.0.mm.3D_glrlm_RunLengthNonUniformityNormalized |
|  | Arterial (8) | original_firstorder_90Percentile |
|  |  | wavelet.HHL_glrlm_LongRunLowGrayLevelEmphasis |
|  |  | original_shape_Sphericity |
|  |  | original_glszm_SmallAreaLowGrayLevelEmphasis |
|  |  | log.sigma.2.0.mm.3D_glcm_Correlation |
|  |  | original_glszm_SmallAreaEmphasis |
|  |  | original_shape_Flatness |
|  |  | wavelet.LLH_firstorder_Minimum |
|  | Venous (7) | original_firstorder_Mean |
|  |  | original_shape_Sphericity |
|  |  | wavelet.LLH_glcm_Correlation |
|  |  | wavelet.HLL_glszm_LargeAreaLowGrayLevelEmphasis |
|  |  | wavelet.LLH_firstorder_Maximum |
|  |  | log.sigma.2.0.mm.3D_glcm_Correlation |
|  |  | wavelet.HLL_glcm_Idn |
|  | Delayed (4) | wavelet.HLL_glcm_Idn |
|  |  | original_firstorder_10Percentile |
|  |  | wavelet.LLL_gldm_LargeDependenceLowGrayLevelEmphasis |
|  |  | wavelet.LHL_glszm_LowGrayLevelZoneEmphasis |
|  | Combined (10) | original_firstorder_Mean_v |
|  |  | log-sigma-2-0-mm-3D_glcm_Correlation_v |
|  |  | wavelet-HHL_glrlm_LongRunLowGrayLevelEmphasis_a |
|  |  | original_shape_Sphericity_a |
|  |  | wavelet-HLL_glszm_LargeAreaLowGrayLevelEmphasis_v |
|  |  | wavelet-LLH_firstorder_10Percentile |
|  |  | log-sigma-2-0-mm-3D_glcm_Correlation_a |
|  |  | original_shape_Sphericity_v |
|  |  | original_glszm_SmallAreaLowGrayLevelEmphasis_a |
|  |  | original_glszm_SmallAreaEmphasis_a |

Note: _a, indicates that this feature is originated from the arterial phase; _v, indicates that this feature is originated from the venous phase; _d, indicates that this feature is originated from the delayed phase.
